# Supplementary material for: The multisystemic roots of South African child and youth resilience: A scoping review
Source: PLoS One. 2025 Nov 14;20(11):e0336716. doi: 10.1371/journal.pone.0336716 (PMC12617933; doi:10.1371/journal.pone.0336716)
Supplement: S1 Table — (DOCX) [file pone.0336716.s002.docx]

## Table 2. Profile of South African child and youth resilience studies, 2018-2023

| **Citation** | **Design** | **Sample^[[1]](#footnote-1)^** | **Risk** | **Outcomes** |
| --- | --- | --- | --- | --- |
| Adefehinti and Arts [1] | Qualitative interviews | 18, 94%m, 11-17 (NR) | Unaccompanied migration | Survival (purposively selected to illustrate their “survival instincts”) |
| Adigun and Ndwandwe [2] | Quantitative survey | 292, 56%m, 13-20 (17) | Deafness during COVID | Academic resilience in online learning (as measured by the Academic Resilience Scale, ARS-30, Cassidy 2016) |
| Asante, Meyer-Weitz [3] | Quantitative survey | 207, 77%f, 17-20 (NR) | Life transition from school into first-year university | Productive coping (as measured by the Adolescent Coping Scale, ACS, Frydenberg & Ramon, 1993) |
| Bain and Durbach [4] | Qualitative interviews | 6, 100%f, 20-25 (NR) | Lived with parents or stepparents involved in IPV for extended periods during childhood | Secure attachment states of mind (based on the Adult Attachment Interview, AAI, George et al., 1996) |
| Berejena Mhongera and Lombard [5] | Qualitative visual methods | 5, 60%m, 13-18 (NR) | Socioeconomic adversity | Coping well with adverse situations (self-reported via drawings) |
| Bezuidenhout, Theron [6] | Qualitative visual methods | 1, female, 7 | Parental divorce & transition into Grade 1 | ‘Competence’ or adjustment to Grade 1 (judged by a panel of 2 psychologists and 3 teachers) |
| Bireda and Pillay [7] | Qualitative visual methods | 10, 60%f, 12-14 (NR) | Orphaned by AIDS | Positive child behavior (social worker completed checklist), good academic and school conduct. |
| Bodiroa and Ross [8] | Qualitative interviews | 12, 58%m, 18-21 (NR) | Transitioning out of state care to independent living  Most had ‘special needs’ such as mental health conditions, behavioral problems or were undocumented | Independent living (8 participants reported living independently, 3 with family, 1 with boyfriend) |
| Bond and Van Breda [9] | Qualitative visual methods | 12, NR, 17-18 (NR) | (Aging out of) residential care | Positive future self; self-reported |
| Botha and van der Merwe [10] | Mixed methods | 500, 61%f, NR (21) | Studying medicine | Avoiding burnout (Copenhagen Burnout Inventory, Kristensen et al., 2005) and depression, resilience (self-reported as high or low) |
| Burger and Naude [11] | Qualitative focus groups | 26, 58%f, 81% under 23, 19% 23 or older (NR) | Life transition from school into first-year university | Academic success – achievement, goal attainment, feelings of satisfaction, and application of new skills (defined by focus groups) |
| Christodoulou, Rotheram-Borus [12] | Quantitative longitudinal (6 waves) | 1073, NR, NR (5) | Raised in an informal settlement / township | Healthy at all their reported assessments (growth, cognition & behavior), reflecting a time-independent, dichotomous variable (yes/no) over the 5-year assessment period |
| Crombie, Crombie [13] | Quantitative survey | 443, 72.9%f, NR (NR)  Students in 2^nd^ to 6^th^ year of study | Medical students’ experiences of mistreatment by clinicians and academics at a South African university | No/minimal psychological distress (K10) |
| Crowley, van der Merwe [14] | Quantitative survey | 385, 58%f, 13-18 (median 15) | HIV-related stigma  Stressful life events | Viral suppression (blood tests)  Behavioral resilience (as measured by the Strengths and Difficulties Questionnaire, SDQ, Goodman, 1997) |
| Daniels, Struthers [15] | Qualitative focus groups | 35, 97%m, 18-25 (NR) | Homophobia and discrimination | Educational attainment – completing high school (self-reported) |
| De Jager [16] | Qualitative visual methods | 20, NR, NR (NR)  University students | COVID-19 | Wellbeing (self-reported) |
| Dekel and van Niekerk [17] | Qualitative interviews | 7, 100%f, 19-45 (27) | Burn  Limited financial and structural resources | Postburn coping (self-reported) |
| Dietrich, Jonas [18] | Quantitative survey | 568, 100%f, 15-24 (NR) | HIV | Knowledge of HIV status (self-reported)  ART exposure (blood test) |
| Donald, Wedderburn [19] | Quantitative survey | 734, 52%m, 2 (2) | Low socio-economic status community, with risks for depression, childhood trauma, IPV and poverty | Child development (developmental assessments conducted by trained assessors blinded to background, using the Bayley-III Scales of Infant and Toddler Development (BSID-III)) |
| Du Toit, van der Linde [20] | Quantitative survey | 276, 55%f, 3-11 (4) | Low-income community  Family exposure to an average of five environmental and/or biological risks  80% of children had developmental delays | No developmental delay (Vineland Adaptive Behavior Scales, Third Edition (Vineland-3), Sparrow et al., 2016) |
| Duby, Bunce [21] | Mixed methods | 515, 100%f, 15-24 (NR) | COVID-19  Mental health stressors and vulnerabilities | Mental health (measured with a set of 10 quantitative items) |
| Duby, Jonas [22] | Mixed methods | 515, 100%f, 15-24 (NR) | COVID-19 lockdowns and school closures  Low socio-economic status  Lack of cell phone access  Household food insecurity  High HIV prevalence  High rates of teenage pregnancy | Educational resilience (53% of those enrolled in education prior to COVID-19 (beginning of 2020) were still enrolled in October 2020) |
| Duma [23] | Qualitative interviews | 7, 86%f, NR (NR)  University students | Disability (wheelchair users or dwarfism) | Continued academic performance (self-reported) |
| Filiatreau, Giovenco [24] | Quantitative survey | 334, 71%f, 16-23 (21) | Physical and sexual violence | Absence of depressive symptoms (Center for Epidemiological Studies – Depression  Scale, Radloff, 1977) |
| Francis, Myers [25] | Quantitative survey | 20227, 55.4%f, 10-23 (NR) | Adolescence: “a period of experimentation and heightened risk of engaging in risky behaviours” | No/minimal alcohol, tobacco or cannabis use.  No/minimal risky sexual behavior.  (self-reported in survey) |
| Gerrand and Nduna [26] | Qualitative interviews | 15, 100%f, 15-18 (NR) | Father absence  Living in a community with high HIV prevalence, high unemployment and poverty, and low economic growth | Coping with life  without the presence of a father (self-report)  Healthy psychological and emotional development of youth (self-report) |
| Gibbs, Jewkes [27] | Quantitative survey | 1357, 50%f, NR (~24 years) | Living in an informal settlement  Not in formal employment  Food insecurity  Childhood trauma  Substance misuse  Poor mental health | No/minimal IPV – experienced by women, perpetrated by men (modified WHO Violence Against Women (VAW) scale, Jewkes et al., 2006) |
| Gordon, Martin [28] | Quantitative survey | 1305, 82%f, 15-24 (NR) | Sexual and reproductive health risks, including PrEP discontinuation | No/minimal depression (Patient Health Questionnaire-9, Spitzer et al., 1999) |
| Groves, Gebrekristos [29] | Mixed methods | 109, 100%f, 14-19 (17) | Drop out of school | Return to school (single item, self-report) |
| Haag, Du Toit [30] | Quantitative longitudinal (2 waves) | 233, 55%f, 19-21 (20) | COVID-related changes in mental health | No/minimal depression (Patient Health Questionnaire 9, Kroenke et al., 2001), anxiety (Generalized Anxiety Disorder 7, Spitzer et al., 2006) and alcohol use (Alcohol Use Disorders Identification Test, Saunders et al., 1993) |
| Haffejee and Wiebesiek [31] | Qualitative case study | 1, female, 19 | Becoming a trans girl in a rural community | Owning her trans identity (self-reported) |
| Hemson [32] | Qualitative case study | 4, 100%m, NR (NR)  High school age | Severe financial, social and educational constraints | Exceptional academic results (Matric marks) |
| Hlungwani and van Breda [33] | Qualitative interviews | 9, 100%f, 21-27 (NR) | Care-leavers; transitioning to adulthood | Functional behavior (“not be doing ‘too badly’, for example, not taking drugs or doing crime” p. 918) |
| Hlungwani and van Breda [34] | Qualitative interviews | 9, 55%m, NR (NR)  Adults reporting on youth | Residential care; transitioning to adulthood | Successful transitioning out of residential care |
| Höltge, Ungar [35] | Quantitative survey | 599, 65%f, 14-24 (18) | Severe parental conflict | Mental health (low scores on Beck Depression Inventory-II, Beck et al., 1996) |
| Humm, Kaminer [36] | Quantitative survey | 615, NR, 12-15 (13) | Violence exposure  Low-income community | No/minimal mental health: depression (Social and Health Assessment Scales, Rushkin et al., 2004), aggression (Aggression Questionnaire, Buss & Perry, 1992), conduct disorder (Child Behaviour Checklist, Achenbach & Edelbrock, 1983) |
| Khumalo, Van Staden [37] | Qualitative interviews | 9, 55.5%m, NR, ~18  Final year of high school | Disadvantaged learners in a disadvantaged community – harmful, non-conducive, non-supportive home environments for learning mathematics | Mathematical literacy (“showed some improvement in their mathematics achievement by attaining at least 30% in mathematics performance”, participants recommended by educators as demonstrating mathematical resilience – improvements in marks for mathematics) |
| Kuo, LoVette [38] | Quantitative survey | 195, 56%f, 13-15 (14) | Affected by HIV (living with HIV, caregivers living with HIV, experienced orphanhood) | Fewer behavioral problems (as measured by the Strengths and Difficulties Questionnaire [SDQ]) |
| LoVette, Sullivan [39] | Quantitative survey | 7237, 100%f, 15-24 (19) | Growing up in context fraught with socioecological risk to sexual health | Non-engagement in transactional sex; delayed age (>15) of sexual debut; condom use assessed via self-report questions in survey |
| LoVette, Sullivan [40] | Quantitative, survey | 7237, 100%f, 15-24 (19) | Growing up in context fraught with socioecological risk to sexual health | Non-engagement in transactional sex and delayed age (>15) of sexual debut, assessed via self-report questions in survey |
| Macedo, Sherr [41] | Quantitative longitudinal (2 waves) | 833, NR, 4-13 (NR) | Parental loss/ orphanhood (reported at baseline) | Sustained mental health resilience (i.e., absence of mental health problems at both baseline and follow-up; measured via self- and caregiver-report using items from validated scales (e.g., SDQ, CDI) |
| Machenjedze, Malindi [42] | Qualitative visual methods | 23, 61%f, 13-17 (NR) | AIDS-related orphanhood and vulnerability (self-reported) | Coping with life when life is hard (self-reported resources) |
| Malindi [43] | Qualitative interviews & focus group | 10, 100%f, 16-19 (NR) | Teenage motherhood | Coping [well] with teenage motherhood and return to school (self-reported) |
| Malindi and Hay [44] | Qualitative visual methods | 20, 55%f, 12-19 (NR) | In care; COVID-19 pandemic (sampled from group home care institution during the pandemic) | Coping well with COVID-19 challenges (self-reported) |
| Mohangi [45] | Qualitative case study | 5 grandparents, 7 parents, 4 children, 9-15 (NR) | Resource-constrained multigenerational households (sampled from); COVID-19 pandemic (Sampled during) | Positive child development, child wellbeing (self-reported) |
| Mostert and Castello [46] | Quantitative survey | NR | Poverty | Better education outcomes and spillover effects for parental employment |
| Motsabi, Diale [47] | Mixed methods | 311, NR%, NR (NR)  11- & 14-year-old cohorts | Challenges of retention and throughput amongst first-year first-generation African students | Academic persistence (interview participants had persisted through the first semester and passed all their first semester modules in 2014) |
| Motsabi, Diale [48] | Qualitative interviews & focus groups | 23, 56%f, 18-20 (NR) | Challenges of retention and throughput amongst first-year first-generation African students | Academic success (passed first semester or first year classes, and did not drop out) |
| Mukuna [49] | Qualitative case study | 4, 100%m, 17-20 (NR) | Early parenthood | Resilient fatherhood (self-reported, continued schooling) |
| Mukuna and Aloka [50] | Qualitative case study | 10, 100%f, 16-20 (NR) | Teenage motherhood | Resilient teenage motherhood (self-reported; continued schooling) |
| Nadat and Jacobs [51] | Qualitative interviews | 14, 100%f, 18-25 (NR) | Structural adversity; psychosocial risk (inferred from residence in a township) | Altruism (engaged in volunteer work for at least 1 year) and continued education (sample are alumni from a youth development organization who volunteer to teach learners on Saturday mornings; 53% of alumni are engaged in tertiary studies) |
| Naidoo, Muthukrishna [52] | Qualitative interviews | 7, 100%f, NR (NR)  All in grades 11-12; became mothers at 15-17 years | Teenage motherhood | Continued education (following teenage motherhood) |
| Nyati, Patel [53] | Quantitative survey | 162, NR, NR (NR)  Grades R & 1 | Growing up in low-income household (all recipients of child support grant) | Child wellbeing (assessed with self-developed child wellbeing tracking tool); physical growth (height and weight measurements taken by nurses); resilience (assessed via CYRM) |
| Padmanabhanunni and Pretorius [54] | Quantitative survey | 337, 77%f, NR (22) | COVID-19 pandemic and related loneliness | Life satisfaction (measured by Satisfaction with Life Scale) |
| Padmanabhanunni and Pretorius [55] | Quantitative survey | 337, 77%f, NR (22) | COVID -19 pandemic and related loneliness | No/minimal levels of loneliness (measured by UCLA Loneliness Scale) |
| Padmanabhanunni and Pretorius [56] | Quantitative survey | 337, 77%f, 16-28 (22) | COVID-19-related psychological distress (hopelessness & depression) | Life satisfaction (measured by Satisfaction with Life Scale) and psychological wellbeing |
| Parchment and Small [57] | Quantitative survey | 577, 59%f, 9-13 (NR) | Early adolescence; high risk of psychological distress and risky sexual behavior in adolescence | No/minimal rates of engagement in risky sexual situations (as measured by Sexual Possibilities Scale) and mental health (no/ltd symptoms of depression as measured by Child Depression Inventory) |
| Pretorius [58] | Quantitative survey | 174 82%f, NR (22) | COVID-19 and related hopelessness and depression | Psychological wellbeing (No/minimal hopelessness and depression) |
| Pretorius and Theron [59] | Qualitative visual methods | 17, 65%f, 17-19 (18) | Township-related risks (structurally inferior; low-income)  High parental expectations (household tasks and scholastics) | Positive adjustment (panel of teachers identified participants who showed “evidence that they were doing well despite township risks”) |
| Reed, Maodzwa-Taruvinga [60] | Quantitative survey | N_SA_ 362 (64%f, NR (23) | Risks of being a first-generation student | University adaptation (using Academic Resourcefulness Inventory and University Adaptation Questionnaire) and academic self-efficacy (using Academic Self-efficacy Measure) |
| Reyes, Maman [61] | Quantitative secondary data | 1089, 100%f, NR (26) | Physical or sexual IPV victimization | No/minimal levels of emotional distress (measured by Hopkins Symptoms Checklist) |
| Romero, Cluver [62] | Quantitative secondary data | 599, NR, 16-18 (NR) | Socioeconomic disadvantage | Being in the age-appropriate grade (measured by school records) |
| Rotheram-Borus, Christodoulou [63] | Quantitative survey | 1073, NR, 2 weeks to 60 months (NR) | Children of mothers living with HIV; children living in low-income townships | Normative physical, cognitive and behavioral development (“Children were considered ‘resilient’ at each assessment if their developmental scores were within two SDs from the standardized mean scores for each variable”; measured using WHO adjusted norms for height; Bayley Scale of Infant Development, Peabody Picture Vocabulary Test, & Mental Processing Index of the Kaufman Assessment Battery for Children; Achenbach’s Child Behavior Checklist & Strengths and Difficulties Questionnaire). |
| Sharp, Penner [64] | Quantitative, survey | 750, 43%f, 7-11 (9) | Being orphaned | Mental health (as assessed by the Strengths and Difficulties Questionnaire) |
| Shenderovich, Boyes [65] | Quantitative longitudinal (3 waves) | 926, 55%f, 10-19 (14) | Adolescents living with HIV | Mental wellbeing (Low depression and anxiety scores; measured using Child Depression Inventory short form and Children’s Manifest Anxiety Scale – Revised) |
| Sherr, Yakubovich [66] | Quantitative longitudinal (2 waves) | Children attending CBOs 383, 51%f, 4-13 (11); Young carers 1358, 56%f, 9-18 (12) | Structural disadvantage; family adversity | No/minimal depression (measured using Children’s Depression Inventory) |
| Singh and Naicker [67] | Qualitative interviews | 16, 100%f, 16-20 (NR) | Teenage motherhood, poverty | Return to school after giving birth and persisting in education |
| Somefun, Theron [68] | Quantitative longitudinal (2 waves) | 914, 57%f (wave 1) and 528, 61%f (wave 2), 14-24 (18) | Family adversity, community economic instability, or community violence | Better mental health outcomes: conduct problems (Delinquency Scale, Geldhof et al., 2014) and depression (Beck Depression Inventory-II (Beck et al., 1996) |
| Sui, Massar [69] | Qualitative interviews | 30, 57%m, 14-19 (NR) | Violence exposure (by proxy of residence in violent Cape Town neighborhoods; all participants reported direct or indirect experience of violence) | Positive psychological and behavioral functioning (by implication, self-reported) |
| Themane and Mabasa [70] | Qualitative case study | 18, NR, NR (NR)  Undergraduate students | COVID-19 disruptions to education; historic disadvantage (inclusion criteria: NSFAS recipients only with township backgrounds and schooling in quintile 1-3 schools) | Coping successfully with challenges to learning during the pandemic (inclusion criterion: “they had to have succeeded in their studies, and not to have repeated a subject in their studies”) |
| Theron [71] | Qualitative visual methods | 385 youth, 59%f, 11-18 (13.8)  284 adults, 64%f, 21-69 (41) | Structural disadvantage | Youth: “to do well in your life so far (even though life is difficult)” (self-report)  Adults: “helped youth in your community to do well when they lead difficult lives” (self-report) |
| Theron and van Rensburg [72] | Qualitative longitudinal visual methods (2 waves) | 140, 62%f, NR (14 at T1) | Living in a community in economic recession, with low levels of school completion, high unemployment and lack of safety | To do well in life (self-defined & self-reported) |
| Theron and van Rensburg [73] | Qualitative secondary data (visual methods) | Rural: 133, 62%f, NR (16); Urban: 385, 58%f, NR (14) | Living in structurally disadvantaged communities, vulnerable to unemployment, poverty, overcrowding, poor infrastructure, limited health and welfare services | Academic progress at school (rural participants judged as resilient by community-based gate keepers, at least in part because of making progress at school; urban participants judged as resilient because progressed to high school despite relentless structural barriers) |
| Theron, Höltge [74] | Mixed methods longitudinal (3 waves; qualitative visual methods) | 223, 65%f, 15-23 (17) | Family adversity, victimization by community, or neighborhood or community risk | No/minimal depression (Beck Depression Inventory-II, Beck et al., 1996) |
| Theron, Levine [75] | Qualitative visual methods | 16, 56%m, 18-24 (22) | Structural disadvantage and associated risks | “To be strong/do okay when life is hard” (self-report) |
| Theron, Levine [76] | Qualitative longitudinal (5 waves) | 24, 58%f, 18-24 (20) | COVID-19 and related challenges (e.g., lockdown, education, employment, knowing someone with or who died from COVID-19) | Manage later COVID risks (self-reported) |
| Theron, Levine [77] | Qualitative secondary data | 24, 58%f, 18-24 (20) | COVID compliance being threatened by forgetfulness; preventive measures conflicting with personal/collective style; and structural constraints | Compliance with COVID-related public health measures (self-reported) |
| Theron, Mampane [78] | Qualitative secondary data (visual methods) | 25, 64%f, 15-18 (16) | Stressors associated with drought (e.g., poverty and social inequity) and structurally disadvantaged community | Physical and mental health (self-reported) |
| Theron, Murphy [79] | Qualitative case study | 21, 62%m, 17-23 (20) | Oil-dependent communities, vulnerable to unemployment risk, poverty, and youth mental health challenges | Health and wellbeing (self-reported) |
| Theron, Rothmann [80] | Quantitative survey | 576, 55%f, 14-24 (NR) | Oil-dependent communities, vulnerable to unemployment risk, poverty, and youth mental health challenges | No/minimal depression (Beck Depression Inventory-II, Beck et al., 1996)  No/minimal conduct disorder (Enactment of Violence Scale, Geldhof et al., 2014) (no significant predictors) |
| Theron, Rothmann [81] | Quantitative survey | 891, 56%f, 13-24 (NR) | Chronic structural disadvantage | No/minimal depression (Beck Depression Inventory-II; Beck et al., 1996)  No/minimal conduct problems (Enactment of Violence Scale; Geldhof et al., 2014) |
| Theron, Ungar [82] | Mixed methods (qualitative visual methods) | 233, 69%f, 18-29 (25) | Disadvantaged community (e.g. high density, poor infrastructure, and low socioeconomic status), family adversity or community-related stress | No/minimal depression (Beck Depression Inventory-II; Beck et al., 1996) |
| Theron, Ungar [83] | Quantitative longitudinal (2 waves) | 172, 66%f, NR, (18) | COVID-related disruptions to schooling | School engagement (School Engagement Scale, Lam et al., 2014) |
| Theron, Ungar [84] | Mixed methods longitudinal (2 waves) | 172, 66%f, 14-23 (16) | Living in communities that rely on the volatile oil and gas industry | School engagement trajectories (School Engagement Scale, Lam et al., 2014) |
| Van Breda [85] | Quantitative survey | 116 children, 53%f, 6-15 (10); 118 family members, NR, NR (NR) | Family vulnerability | Academic achievement (measured using the One Minute Reading Test and the Schonell Spelling Test) |
| Van Breda [86] | Quantitative longitudinal (8 waves) | 100, 79%m, NR (NR)  Median age 18 at disengagement from care | Leaving care; transitioning into adulthood | Positive independent living outcomes measure (19 care-leaving outcomes; e.g., self-supporting  accommodation, criminal activity and well-being)”. |
| Van Breda [87] | Quantitative survey | 232, 58%f, NR (22) | Transition to university; 1^st^ year students with background of psychosocial vulnerability | Satisfaction with life (using Satisfaction with Life Scale) and academic progress (measured via number of modules failed, passed with distinction and student subjective rating of their progress) |
| Van Breda and Hlungwani [88] | Qualitative interviews | 9, 100%f, 21-27 (NR) | Leaving care; transitioning into adulthood | In the judgement of the children’s homes, doing ‘reasonably well’ since leaving care (e.g. who were not involved in crime or drugs at the time of data collection |
| Van der Merwe, Botha [89] | Quantitative survey | 500, 62%f, NR (20 preclinical, 23 clinical) | Stressful demands of completing a medical degree (high academic workload, emotional and physical challenges) | No/minimal levels of burnout (as measured by Copenhagen Burnout Inventory) |
| Van der Wal and George [90] | Quantitative survey | 962, 58%f, NR (16) | Risk of self-harm during adolescence | No/minimal risk of self-harm (self-reported; single item asking if students ever engaged in self-harm) |
| Van Rensburg, Theron [91] | Quantitative survey | 407, 52%f, 13-19 (16) | Adolescents living in a district experiencing multiple daily risks related to high unemployment, violent crime, poor infrastructure, poverty, poor service delivery, and HIV/AIDS | Resilient: youth capacity to be future-oriented, value-driven, invested in education, and stoical, and with youth access to an active support network – the advisory panel’s definitional criteria for the ‘resilient’ group |
| Van Rensburg, Theron [92] | Quantitative survey | 730, 53%f, 12-19 (16) | Lack of community safety, negative peer support, poor relationship with caregiver(s), and antisocial, health risk, and/or disruptive behaviors | School attendance (4 items from the National Longitudinal Survey of Children and Youth, Resilience Research Centre, 2010) |
| Van Wyk, Mason [93] | Quantitative survey | 248 (71% male), NR (20) | First year students: Life transition from school into first-year university | Academic success (operationalized as academic performance and turnover intentions) |
| Wakefield and Theron [94] | Qualitative visual methods | 302, 59%f, 15-23 (17) | Living in a community characterized by transmissible illnesses, poor employment opportunities, poor infrastructure, rapid population growth rates, high levels of unemployment, and cramped living spaces during the COVID-19 lockdown | To do well or be OK when life was hard (self-reported) |
| Wegner, Stirrup [95] | Qualitative interviews | 65, 65%f, 18-32 (22.2) | Lockdown and COVID-19 challenges | Persistent engagement in leisure activities under COVID-19 |
| Wills and Hofmeyr [96] | Quantitative secondary data | 2383, NR, NR (NR)  Grade 4 learners | Poverty | Academic resilience (measured via comprehension tests used in the PIRLS assessment) |
| Winberg, Winberg [97] | Quantitative secondary data | 2 case studies, 50%f, NR (NR)  Undergraduate students | Transfer-related challenges | Academic persistence (comparing transfer and non-transfer students’ course grades, attrition, transfer out of ECM programs, academic exclusions, retention, and graduation rate) |
| Yang and Wild [98] | Quantitative survey | 536, 58%f, 13-15 (14) | Disruptive family events | Psychological adjustment (measured using Strengths and Difficulties Questionnaire), e.g., peer and conduct problems, hyperactivity |
| Zanoni, Archary [99] | Qualitative interviews | 41, 54%m, 14-16 (NR) | Challenges of living with HIV, including disengagement from care | Engagement in care and good health (“Majority were able to stay engaged in care (98%) and remain virally suppressed (93%) at the time of enrolment”) |

**References**

1. Adefehinti B, Arts K. Challenging the odds of vulnerability and resilience in lone migration: coping strategies of Zimbabwean unaccompanied minors in South Africa. Children’s Geographies. 2019;17(4):427-41. doi: 10.1080/14733285.2018.1536776

2. Adigun OT, Ndwandwe ND. Academic resilience among deaf learners during e-learning in the COVID-19 era. Research in Social Sciences and Technology. 2022;7(2):27-48. doi: 10.46303/ressat.2022.8

3. Asante KO, Meyer-Weitz A, Okafo DC. Psychological carital and orientation to happiness as protective factors for coping among first year university students in South Africa. Int J Health Promot Educ. 2022;60(5):298-307. doi: 10.1080/14635240.2022.2065513

4. Bain K, Durbach C. Adaptation, resilience, and secure attachment states of mind in young South African female students exposed to intimate partner violence in childhood. Journal of Interpersonal Violence. 2021;36(5):2541-71. doi: 10.1177/0886260518759652

5. Berejena Mhongera P, Lombard A. Pathways of resilience for children facing socio-economic adversities: Experiences from Future Families' OVC programmes in South Africa. Children & Youth Services Review. 2020;108:104657. doi: 10.1016/j.childyouth.2019.104657

6. Bezuidenhout C, Theron L, Fritz E. Positive adjustment to first grade despite divorce: Lessons for school psychologists. School Psychology International. 2018;39(5):490-509. doi: 10.1177/0143034318791332

7. Bireda AD, Pillay J. Enhancing protective factors in South African adolescents affected by HIV/AIDS. Vulnerable Children and Youth Studies. 2018;13(2):183-94. doi: 10.1080/17450128.2017.1395101

8. Bodiroa A, Ross E. Challenges, agency and ecology in the transition of youth from state residential care to independent living in Johannesburg, South Africa. Child & Youth Services. 2023;44(3):328-54. doi: 10.1080/0145935X.2022.2113871

9. Bond S, Van Breda AD. Interaction between possible selves and the resilience of care-leavers in South Africa. Children and Youth Services Review. 2018;94:88-95. doi: 10.1016/j.childyouth.2018.09.014

10. Botha A, van der Merwe LJ. 'I am still here!' Undergraduate medical students’ perceptions of resilience. Journal of Psychology in Africa. 2019;29(6):589-97. doi: 10.1080/14330237.2019.1689463

11. Burger A, Naude L. In their own words: Students’ perceptions and experiences of academic success in higher education. Educational Studies. 2020;46(5):624-39. doi: 10.1080/03055698.2019.1626699

12. Christodoulou J, Rotheram-Borus MJ, Rezvan PH, Comulada WS, Stewart J, Almirol E, et al. Where you live matters: Township neighborhood factors important to resilience among South African children from birth to 5 years of age. Preventive Medicine. 2022;157:106966. doi: 10.1016/j.ypmed.2022.106966

13. Crombie KE, Crombie KD, Salie M, Seedat S. Medical students’ experiences of mistreatment by clinicians and academics at a South African university. Teaching and Learning in Medicine. 2023:Advance Online Publication. doi: 10.1080/10401334.2023.2167207

14. Crowley T, van der Merwe AS, Esterhuizen T, Skinner D. Resilience of adolescents living with HIV in the Cape Metropole of the Western Cape. AIDS Care. 2022;34(9):1103-10. doi: 10.1080/09540121.2021.1961115

15. Daniels J, Struthers H, Maleke K, Catabay C, Lane T, McIntyre J, et al. Rural school experiences of South African gay and transgender youth. Journal of LGBT Youth. 2019;16(4):355-79. doi: 10.1080/19361653.2019.1578323

16. De Jager S. Connection, desperation and disillusionment: Exploring student wellbeing at a university in South Africa during the COVID-19 pandemic. Perspectives in Education. 2023;41(1):38-55. doi: 10.38140/pie.v41i1.6140

17. Dekel B, van Niekerk A. Women's recovery, negotiation of appearance, and social reintegration following a burn. Burns. 2018;44(4):841-9. doi: 10.1016/j.burns.2017.12.004

18. Dietrich JJ, Jonas K, Cheyip M, Appollis TM, Ariyo O, Beauclair R, et al. Examining the relationship between psychosocial factors with knowledge of HIV‐positive status and antiretroviral therapy exposure among adolescent girls and young women living with HIV in South Africa. AIDS and Behavior. 2023;27(1):231-44. doi: 10.1007/s10461-022-03759-6

19. Donald KA, Wedderburn CJ, Barnett W, Nhapi RT, Rehman AM, Stadler JAM, et al. Risk and protective factors for child development: An observational South African birth cohort. PLoS Medicine. 2019;16(9):e1002920. doi: 10.1371/journal.pmed.1002920

20. Du Toit M, van der Linde J, Swanepoel DW. Early childhood development risks and protective factors in vulnerable preschool children from low-income communities in South Africa. Journal of Community Health. 2021;46(2):304-12. doi: 10.1007/s10900-020-00883-z

21. Duby Z, Bunce B, Fowler C, Bergh K, Jonas K, Dietrich JJ, et al. Intersections between COVID-19 and socio-economic mental health stressors in the lives of South African adolescent girls and young women. Child and Adolescent Psychiatry and Mental Health. 2022;16(23). doi: 10.1186/s13034-022-00457-y

22. Duby Z, Jonas K, Bunce B, Bergh K, Maruping K, Fowler C, et al. Navigating education in the context of COVID-19 lockdowns and school closures: Challenges and resilience among adolescent girls and young women in South Africa. Frontiers in Education 2022;7:856610. doi: doi:10.3389/feduc.2022.856610

23. Duma PT. Diversity includes disability: Experiences of resilience in a university residence. Journal of Student Affairs in Africa. 2019;7(2):75-87. doi: 10.24085/jsaa.v7i2.3826 75

24. Filiatreau LM, Giovenco D, Twine R, Gómez-Olivé FX, Kahn K, Haberland N, et al. Examining the relationship between physical and sexual violence and psychosocial health in young people living with HIV in rural South Africa. Journal of the International AIDS Society. 2020;23(12):e25654. doi: 10.1002/jia2.25654

25. Francis JM, Myers B, Nkosi S, Petersen Williams P, Carney T, Lombard C, et al. The prevalence of religiosity and association between religiosity and alcohol use, other drug use, and risky sexual behaviours among grade 8-10 learners in Western Cape, South Africa. PloS ONE. 2019;14(2):e0211322. doi: 10.1371/journal.pone.0211322

26. Gerrand P, Nduna M. Father absence in the lives of female African youth living in Mpumalanga, South Africa: Christianity a coping strategy that builds and strengthens resilience. Social Work & Christianity. 2021;48(2):183-99. doi: 10.34043/swc.v48i2.189

27. Gibbs A, Jewkes R, Willan S, Washington L. Associations between poverty, mental health and substance use, gender power, and intimate partner violence amongst young (18- 30) women and men in urban informal settlements in South Africa: A cross-sectional study and structural equation model. PLoS ONE. 2018;13(10):e0204956. doi: 10.1371/journal.pone.0204956

28. Gordon KJ, Martin CE, Kutywayo A, Cox LA, Nongena P, Mullick S. Mental health needs of adolescent and young adult PrEP users in South Africa: Implications for sexual and reproductive health programming. Journal of Adolescent Health. 2023;73(6):S73-S80. doi: 10.1016/j.jadohealth.2023.08.018

29. Groves AK, Gebrekristos LT, McNaughton Reyes L, Moodley D, Raziano V, Maman S. A mixed-methods study of resilience and return to school among adolescent mothers in South Africa. Global Public Health. 2022;17(9):2111-24. doi: 10.1080/17441692.2021.1970208

30. Haag K, Du Toit S, Skeen S, Roberts KS, Chideya Y, Notholi V, et al. Predictors of COVID-related changes in mental health in a South African sample of adolescents and young adults. Psychology, Health & Medicine. 2022;27(sup1):239-55. doi: 10.1080/13548506.2022.2108087

31. Haffejee S, Wiebesiek L. Resilience and resistance: The narrative of a transgender youth in rural South Africa. Gender Issues. 2021;38(3):344-60. doi: 10.1007/s12147-021-09285-4

32. Hemson C. Agency, resilience and innovation in overcoming educational failure. Perspectives in Education. 2018;36(2):61-74. doi: 10.18820/2519593X/pie.v36i2.6

33. Hlungwani J, van Breda AD. Female care leavers' journey to young adulthood from residential care in South Africa: Gender‐specific psychosocial processes of resilience. Child & Family Social Work. 2020;25(4):915-23. doi: 10.1111/cfs.12776

34. Hlungwani J, van Breda AD. Affording managed opportunities for independence to build looked-after young people's resilience: Perceptions and experiences of care workers. Journal of Children's Services. 2022;17(2):137-51. doi: 10.1108/JCS-10-2021-0044

35. Höltge J, Ungar M, Theron L. The differential interplay of home routines and comforting beliefs on adolescent mental health in situations of severe parental conflict. Adversity and Resilience Science. 2021;2(1):5-17. doi: 10.1007/s42844-021-00029-7

36. Humm A, Kaminer D, Hardy A. Social support, violence exposure and mental health among young South African adolescents. Journal of Child and Adolescent Mental Health. 2018;30(1):41-50. doi: 10.2989/17280583.2018.1476358

37. Khumalo VL, Van Staden S, Graham MA. Weathering the storm: Learning strategies that promote mathematical resilience. Pythagoras. 2022;43(1):a655. doi: 10.4102/pythagoras.v43i1.655

38. Kuo C, LoVette A, Pellowski J, Harrison A, Mathews C, Operario D, et al. Resilience and psychosocial outcomes among South African adolescents affected by HIV. Aids. 2019;33(sup1):S29-S34. doi: 10.1097/QAD.0000000000002177

39. LoVette A, Sullivan A, Kuo C, Operario D, Harrison A, Mathews C. Examining associations between resilience and sexual health among South African girls and young women living with and without HIV. AIDS Education and Prevention. 2023;35(1):1-13. doi: 10.1521/aeap.2023.35.1.1

40. LoVette A, Sullivan A, Operario D, Kuo C, Harrison A, Matthews C. Social resources, resilience, and sexual health among South African adolescent girls and young women: findings from the HERStory study. Culture, Health and Sexuality. 2023;25(7):929-43. doi: 10.1080/13691058.2022.2108501

41. Macedo A, Sherr L, Tomlinson M, Skeen S, Roberts K. Parental bereavement in young children living in South Africa and Malawi: Understanding mental health resilience. Journal of Acquired Immune Deficiency Syndromes. 2018;78(4):390-8. doi: 10.1097/qai.0000000000001704

42. Machenjedze N, Malindi MJ, Mbengo F. The feasibility of the draw-and-write technique in exploring the resilience of children orphaned by AIDS. African Journal of AIDS Research. 2019;18(1):72-80. doi: 10.2989/16085906.2018.1556170

43. Malindi MJ. Personal and socio-ecological resilience resources among school-going teenage mothers: An exploratory study. Journal of Psychology in Africa. 2018;28(4):340-3. doi: 10.1080/14330237.2018.1501883

44. Malindi MJ, Hay J. Resilience anchors for children in an out-of-home care institution during and after COVID-19. Frontiers in Psychology. 2023;14:1189739. doi: 10.3389/fpsyg.2023.1189739

45. Mohangi K. Risks and opportunities for children’s well-being in resource constrained multigenerational households during COVID-19: Implications for school psychology interventions. School Psychology International. 2023;44(2):236-54. doi: 10.1177/01430343221144407

46. Mostert CM, Castello JV. Long run educational and spillover effects of unconditional cash transfers: Evidence from South Africa. Economics and Human Biology. 2020;36:100817. doi: 10.1016/j.ehb.2019.100817

47. Motsabi S, Diale B, Van Zyl A. The academic persistence of first‐year first‐generation African students (FYFGAS): A framework for higher education in South Africa. Journal of Student Affairs in Africa. 2020;8(2):73-85. doi: 10.24085/jsaa.v8i2.4449

48. Motsabi S, Diale B, Van Zyl A. The role of social support in the persistence of first-year first-generation African students in a higher education institution in South Africa. South African Journal of Higher Education. 2020;34(4):189-210. doi: 10.20853/34-4-3486

49. Mukuna RK. Exploring Basotho teenage fathers’ experiences of early fatherhood at South African rural high schools. Journal of Psychology in Africa. 2020;30(4):348-53. doi: 10.1080/14330237.2020.1796031

50. Mukuna RK, Aloka PJO. Interpretative phenomenological analysis of teenage mothers resiliency in overcoming adversities in pregnancy and early motherhood in South Africa. Problems of Education in the 21st Century. 2021;79(1):104-17. doi: 10.33225/pec/21.79.104

51. Nadat Y, Jacobs S. Elements that contribute to resilience in young women from a high-risk community. Social Work/Maatskaplike Werk. 2021;57(1):87-100. doi: 10.15270/52-2-908

52. Naidoo J, Muthukrishna N, Nkabinde R. The journey into motherhood and schooling: Narratives of teenage mothers in the South African context. International Journal of Inclusive Education. 2021;25(10):1125-39. doi: 10.1080/13603116.2019.1600053

53. Nyati LH, Patel L, Haffejee S, Sello M, Mbowa S, Sani T, et al. Context matters: Child growth within a constrained socio-economic environment. International Journal of Environmental Research and Public Health. 2022;19:11944. doi: 10.3390/ijerph191911944

54. Padmanabhanunni A, Pretorius TB. The loneliness-life satisfaction relationship: The parallel and serial mediating role of hopelessness, depression and ego-resilience among young adults in South Africa during COVID-19. International Journal of Environmental Research and Public Health. 2021;18(7). doi: 10.3390/ijerph18073613

55. Padmanabhanunni A, Pretorius TB. The unbearable loneliness of COVID-19: COVID-19-related correlates of loneliness in South Africa in young adults. Psychiatry Research. 2021;296:113658. doi: 10.1016/j.psychres.2020.113658

56. Padmanabhanunni A, Pretorius TB. Promoting well-being in the face of a pandemic: the role of sense of coherence and ego-resilience in the relationship between psychological distress and life satisfaction. South African Journal of Psychology. 2023;53(1):124-33. doi: 10.1177/00812463221113671

57. Parchment TM, Small LA. South African child report of caregiver attunement as a mechanism to decrease engagement in risky sexual situations and depression. Vulnerable Children and Youth Studies. 2023;18(1):100-12. doi: 10.1080/17450128.2022.2075066

58. Pretorius T-l. Depression among health care students in the time of COVID-19: The mediating role of resilience in the hopelessness–depression relationship. South African Journal of Psychology. 2021;51(2):269-78. doi: 10.1177/0081246321994452

59. Pretorius T-l, Theron L. 'A pillar of strength’: Empowering women and the resilience of township-dwelling adolescents. YOUNG. 2019;27(4):373-94. doi: 10.1177/1103308818795081

60. Reed M, Maodzwa-Taruvinga M, Ndofirepi ES, Moosa R. Insights gained from a comparison of South African and Canadian first-generation students: The impact of resilience and resourcefulness on higher education success. Compare: A Journal of Comparative and International Education. 2019;49(6):964-82. doi: 10.1080/03057925.2018.1479185

61. Reyes HLM, Maman S, Groves AK, Moodley D. Intimate partner violence and postpartum emotional distress among South African women: Moderating effects of resilience and vulnerability factors. Global Public Health. 2020;15(8):1157-67. doi: 10.1080/17441692.2020.1751233

62. Romero RH, Cluver L, Hall J, Steinert J. Socioeconomically disadvantaged adolescents and educational delay in two provinces in South Africa: Impacts of personal, family and school characteristics. Education as Change. 2018;22(1). doi: 10.25159/1947-9417/2308

63. Rotheram-Borus MJ, Christodoulou J, Hayati Rezvan P, Comulada WS, Gordon S, Skeen S, et al. Maternal HIV does not affect resiliency among uninfected/HIV exposed South African children from birth to 5 years of age. Aids. 2019;33(sup1):S5-S16. doi: 10.1097/QAD.0000000000002176

64. Sharp C, Penner F, Marais L, Skinner D. School connectedness as psychological resilience factor in children affected by HIV/AIDS. AIDS Care. 2018;30(S4):34-41. doi: 10.1080/09540121.2018.1511045

65. Shenderovich Y, Boyes M, Esposti MD, Casale M, Toska E, Roberts KJ, et al. Relationships with caregivers and mental health outcomes among adolescents living with HIV: A prospective cohort study in South Africa. BMC Public Health. 2021;21(1):1-11. doi: 10.1186/s12889-020-10147-z

66. Sherr L, Yakubovich AR, Skeen S, Tomlinson M, Cluver LD, Roberts KJ, et al. Depressive symptoms among children attending community based support in South Africa: Pathways for disrupting risk factors. Clinical Child Psychology and Psychiatry. 2020;25(4):984-1001. doi: 10.1177/1359104520935502

67. Singh S, Naicker P. Development of resilience in teenage mothers within contextual realities of poor rural South Africa. Journal of poverty. 2019;23(7):559-75. doi: 10.1080/10875549.2019.1616038

68. Somefun O, Theron L, Ungar M. The association between family adversity and youth mental health outcomes. Journal of Adolescence. 2023;95(7):1333-47. doi: 10.1002/jad.12205

69. Sui X, Massar K, Reddy PS, Ruiter RAC. Developmental assets in South African adolescents exposed to violence: A qualitative study on resilience. Journal of Child & Adolescent Trauma. 2022;15(1):1-13. doi: 10.1007/s40653-021-00343-3

70. Themane MJ, Mabasa LT. Epistemic access and success of historically disadvantaged students during the COVID-19 pandemic: A South African experience. Perspectives in Education. 2022;40(1):18-38. doi: 10.18820/2519593X/pie.v40.i1.2

71. Theron LC. Adolescent versus adult expectations of resilience enablers: A South African study. Youth & Society. 2020;52(1):78-98. doi: 10.1177/0044118X17731032

72. Theron LC, van Rensburg A. Resilience over time: Learning from school-attending adolescents living in conditions of structural inequality. Journal of Adolescence. 2018;67:167-78. doi: 10.1016/j.adolescence.2018.06.012

73. Theron LC, van Rensburg A. Parent-figures and adolescent resilience: An African perspective. International Journal of School & Educational Psychology. 2020;8(2):90-103. doi: 10.1080/21683603.2019.1657994

74. Theron LC, Höltge J, Ungar M. Multisystemic supports and adolescent resilience to depression over time: A South African mixed methods study. Development and Psychopathology. 2023;35(5):2365-83. doi: 10.1017/S0954579423000494

75. Theron LC, Levine D, Ungar M. African emerging adult resilience: Insights from a sample of township youth. Emerging Adulthood. 2021;9(4):360-71. doi: 10.1177/2167696820940077

76. Theron LC, Levine D, Ungar M. Resilience to COVID-19-related stressors: Insights from emerging adults in a South African township. PLoS ONE. 2021;16(12):e0260613. doi: 10.1371/journal.pone.0260613

77. Theron LC, Levine D, Ungar M. The inhibitors and enablers of emerging adult COVID-19 mitigation compliance in a township context. South African Journal of Science. 2022;118(5):81-8. doi: 10.17159/sajs.2022/13173

78. Theron LC, Mampane MR, Ebersöhn L, Hart A. Youth resilience to drought: Learning from a group of South African adolescents. International Journal of Environmental Research and Public Health. 2020;17(21):7896. doi: 10.3390/ijerph17217896

79. Theron LC, Murphy K, Ungar M. Multisystemic resilience: Learning from youth in stressed environments. Youth & Society. 2022;54(6):1000-22. doi: 10.1177/0044118X211017335

80. Theron LC, Rothmann S, Höltge J, Ungar M. Differential adaptation to adversity: A latent profile analysis of youth engagement with resilience-enabling cultural resources and mental health outcomes in a stressed Canadian and South African community. Journal of Cross-Cultural Psychology. 2022;53(3):403-25. doi: 10.1177/00220221221077353

81. Theron LC, Rothmann S, Makhnach A, Ungar M. Adolescent mental health resilience and combinations of caregiver monitoring and warmth: A person-centred perspective. Journal of Child & Family Studies. 2022;31(10):2860-70. doi: 10.1007/s10826-022-02287-0

82. Theron LC, Ungar M, Cockcroft K, Fouche A. Multisystemic resources matter for resilience to depression: Learning from a sample of young South African adults. Qualitative Health Research. 2023;33(10):828-41. doi: 10.1177/10497323231182906

83. Theron LC, Ungar M, Höltge J. Student resilience to COVID-19-related school disruptions: The value of historic school engagement. School Psychology International. 2023;44(2):190-213. doi: 10.1177/01430343221138785

84. Theron LC, Ungar M, Höltge J. Pathways of resilience: Predicting school engagement trajectories for South African adolescents living in a stressed environment. Contemporary Educational Psychology. 2022;69:102062. doi: 10.1016/j.cedpsych.2022.102062

85. Van Breda AD. Contribution of psychosocial vulnerability and resilience to academic achievement of primary school children in South Africa. Child & Family Social Work. 2022;27(4):679-87. doi: 10.1111/cfs.12916

86. Van Breda AD. The contribution of supportive relationships to care-leaving outcomes: A longitudinal resilience study in south africa. Child Care in Practice. 2022:Advance Online Publication. doi: 10.1080/13575279.2022.2037516

87. Van Breda AD. Resilience of vulnerable students transitioning into a South African university. Higher Education. 2018;75(6):1109-24. doi: 10.1007/s10734-017-0188-z

88. Van Breda AD, Hlungwani J. Journey towards independent living: Resilience processes of women leaving residential care in South Africa. Journal of Youth Studies. 2019;22(5):604-22. doi: 10.1080/13676261.2018.1523541

89. Van der Merwe LJ, Botha A, Joubert G. Burnout and associated factors in undergraduate medical students at a South African university. African Journal of Health Professions Education. 2020;12(2):62-7. doi: 10.7196/ajhpe.2020.v12i2.1172

90. Van der Wal W, George AA. Social support-oriented coping and resilience for self-harm protection among adolescents. Journal of Psychology in Africa. 2018;28(3):237-41. doi: 10.1080/14330237.2018.1475508

91. Van Rensburg AC, Theron LC, Rothmann S. Adolescent perceptions of resilience-promoting resources: The South African pathways to resilience study. South African Journal of Psychology. 2018;48(1):73-85. doi: 10.1177/0081246317700757

92. Van Rensburg AC, Theron L, Rothmann S. A social ecological modeled explanation of the resilience processes of a sample of Black Sesotho-speaking adolescents. Psychological Reports. 2019;122(4):1211-34. doi: 10.1177/0033294118784538

93. Van Wyk M, Mason HD, van Wyk BJ, Phillips TK, van der Walt PE. The relationship between resilience and student success among a sample of south african engineering students. Cogent Psychology. 2022;9:2057660. doi: 10.1080/23311908.2022.2057660

94. Wakefield SE, Theron LC. Exploring Adolescent Resilience during COVID-19 in a South African Township Context. National Youth Advocacy and Resilience Journal. 2023;6(2):48-70. doi: 10.20429/nyarj.2023.060203

95. Wegner L, Stirrup S, Desai H, de Jongh J-C. 'This pandemic has changed our daily living': Young adults’ leisure experiences during the COVID-19 pandemic in South Africa. Journal of Occupational Science. 2022;29(3):323-35. doi: 10.1080/14427591.2022.2078995

96. Wills G, Hofmeyr H. Academic resilience in challenging contexts: Evidence from township and rural primary schools in South Africa. International Journal of Educational Research. 2019;98:192-205. doi: 10.1016/j.ijer.2019.08.001

97. Winberg SL, Winberg C, Engel-Hills P. Persistence, resilience and mathematics in engineering transfer capital. IEEE Transactions on Education. 2018;61(4):281-8. doi: 10.1109/TE.2018.2825942

98. Yang SA, Wild LG. Associations between grandparent involvement and psychological difficulties in adolescents facing family adversity. Journal of Child & Family Studies. 2022;31(5):1489-500. doi: 10.1007/s10826-021-02223-8

99. Zanoni BC, Archary M, Subramony T, Sibaya T, Psaros C, Haberer JE. Disclosure, social support, and mental health are modifiable factors affecting engagement in care of perinatally-HIV infected adolescents: A qualitative dyadic analysis. AIDS and Behavior. 2021;25(1):237-48. doi: 10.1007/s10461-020-02968-1

1. N, % majority gender, age range (age mean); NR = not reported [↑](#footnote-ref-1)
