# Supplementary material for: The multisystemic roots of South African child and youth resilience: A scoping review
Source: PLoS One. 2025 Nov 14;20(11):e0336716. doi: 10.1371/journal.pone.0336716 (PMC12617933; doi:10.1371/journal.pone.0336716)
Supplement: Attachment — Submitted filename: Feedback on revisions 2025.01.15.docx [file pone.0336716.s003.docx]

Dear Editor, thank you for your feedback on our manuscript. We have made the requested revisions. These are set out below:

1. We have checked and corrected conformity to the journal’s manuscript body formatting guidelines
2. We have checked the cover page conformity to the title, author, affiliations formatting guidelines. We have removed our ORCID numbers as the are not mentioned in the guidelines. We have also removed the & from the second author and marked both with ¶, since both authors contributed equally to the paper.
3. We have corrected the labelling of Figures.
4. We have corrected the font size of Level 2 headings to 16pt and Level 3 to 14pt.
5. We have corrected the font size of table headings to 12pt.
6. To reduce the length of the manuscript, we have moved Table 2 into a supplementary file (S1 Table). This brings the manuscript to within 10,000 words as requested.
7. We have aligned Table 1 to the left, rather than centred, for easier reading.
8. We have ensured that all tables and figures appear immediately after they are first referred to in text.
9. We have carefully proofread the manuscript and corrected language errors.
10. We identified a missing space after the publication dates in the reference list and have corrected this.
11. We have checked all manuscripts to ensure they have not been retracted and to make any necessary updates. The following references were changed:
    a. [1] Masten – this advance online publication has since been published
    b. [25] Theron – this advance online publication has since been published
    c. [138] Tamambang – this advance online publication has since been published
12. Two papers in the systematic review were advance online publications when we conducted the search, and have since been published. We have retained the references at the time of the search:
    a. [106] Crombie KE, Crombie KD, Salie M, Seedat S. Medical students’ experiences of mistreatment by clinicians and academics at a South African university. Teaching and Learning in Medicine. 2023:Advance Online Publication. doi: 10.1080/10401334.2023.2167207
    b. [58] Van Breda AD. The contribution of supportive relationships to care-leaving outcomes: A longitudinal resilience study in South Africa. Child Care in Practice. 2022:Advance online publication. doi: 10.1080/13575279.2022.2037516
13. One reference is that is part of the article text, not the review, is still an advance online publication. We have retained its formatting:
    a. [106] Crombie KE, Crombie KD, Salie M, Seedat S. Medical students’ experiences of mistreatment by clinicians and academics at a South African university. Teaching and Learning in Medicine. 2023:Advance Online Publication. doi: 10.1080/10401334.2023.2167207
14. We do recognise that the reference list is substantial. However, we wish to note that 99 of the references are the texts that comprises the scoping review itself. We cannot remove any of these, as these are our core evidence. The remaining 44 references are, in our view, necessary, as the provide the contextual framework for the study overall and particularly for the multisystemic approach to resilience, which, being a recent innovation on resilience science, requires sufficient citation to the scholarship of the field. We thus believe that removing references will harm the integrity and scholarliness of the paper.

Kind regards

Profs Adrian van Breda and Linda Theron
